# T255. WHO PARTICIPATED IN FAMILY WORK IN THE US RAISE-ETP FIRST EPISODE SAMPLE?

**DOI:** 10.1093/schbul/sby016.531

**Published:** 2018-04-01

**Authors:** Shirley Glynn, Susan Gingerich, Piper Meyer-Kalos, Kim Mueser, Alec Chan-Golston, Catherine Sugar, Nina Schooler, John Kane

**Affiliations:** 1 University of California, Los Angeles; 2 Independent Consultant; 3 The University of Minnesota; 4 Boston University; 5 SUNY Downstate Medical Center; 6 Northwell Health

## Abstract

**Background:**

The Recovery After an Initial Schizophrenia Episode Early Treatment Program (RAISE-ETP) is a US NIMH-funded 34 site cluster randomized controlled trial which evaluated the benefits of participation in a multicomponent intervention, entitled NAVIGATE, for first episode psychosis (FEP). Previously, participation in NAVIGATE was reported to yield significant participant benefits, compared to customary care (Kane et al, 2016). NAVIGATE included tailored medication, individual resiliency training, family education, and supported education and employment. Here we examine the absolute rate of family engagement in professional support services in the intent to treat sample, as well identify predictors of participation.

**Methods:**

A total of 404 individuals between ages 15 and 40 were enrolled. DSM-IV diagnoses of non-affective psychosis were included. All participants had experienced only one episode of psychosis, had been prescribed less than 6 months of lifetime psychotic medication, spoke English, and provided informed consent. Participants were offered a minimum of two years of NAVIGATE or customary care (CC). At baseline, participants provided demographic and clinical history information; they were administered the Heinrichs-Carpenter Quality of Life Scale (QOL) and the Positive and Negative Symptom Scale (PANSS). Site research assistants interviewed participants monthly to capture participation in the four types of NAVIGATE interventions, allowing treatment groups to be compared on receipt of key services.

**Results:**

One hundred nineteen of the 404 participants (29.4%) reported their relatives attending five or more family sessions within the first year of randomization (102 families (45.74%) in NAVIGATE; 17 (9.39%) in CC). In a simultaneous logistic regression analysis predicting meeting this five family sessions threshold or not, significant independent predictors (all p <. 05) included treatment group, consumer negative symptoms, consumer self-reported quality of family relationship, race, and consumer residence. Relatives were more likely to attend family sessions if their loved one was 1) randomized to NAVIGATE, 2) had greater negative symptoms on the PANSS, 3) self-reported as emotionally closer to the family, 4) was Caucasian, and 5) lived with family. Other consumer PANSS and QOL scores, consumer age, ethnicity, health insurance status, cigarette smoking status, and consumers’ mother education were not significant independent predictors.

**Discussion:**

Although the benefits of family support and education have been highlighted for persons with a recent onset of psychosis, the results here suggest that engaging relatives in these services, at least in the US, can be challenging. Even given a relatively low threshold of attendance at least 5 family sessions in the first year of treatment, the majority of this sample did not meet the criterion, although participation rates were significantly higher in NAVIGATE. This increase likely reflects the effort NAVIGATE teams expended to engage relatives. It is perhaps not surprising that families of consumers who live with them and/or report feeling closer to them are more likely to attend clinic sessions. Interestingly, higher levels of consumer negative, but not positive symptoms, were also associated with greater attendance at family sessions; this finding suggests that living with a consumer who appears unmotivated and withdrawn may be particularly challenging and prompt relatives to seek more assistance. Finally, our data on race suggest, as other have noted, that greater outreach may be needed to engage non-Caucasian families in services.

